# A cancer-favoring oncolytic vaccinia virus shows enhanced suppression of stem-cell like colon cancer

**DOI:** 10.18632/oncotarget.7660

**Published:** 2016-02-24

**Authors:** So Young Yoo, Seo Young Bang, Su-Nam Jeong, Dae Hwan Kang, Jeong Heo

**Affiliations:** ^1^ BIO-IT Foundry Technology Institute, Pusan National University, Busan 609-735, Republic of Korea; ^2^ Research Institute for Convergence of Biomedical Science and Technology, Pusan National University Yangsan Hospital, Yangsan 626-770, Republic of Korea; ^3^ Department of Internal Medicine, College of Medicine, Pusan National University and Medical Research Institute, Busan 602-739, Republic of Korea; ^4^ Republic of Korea Research Institute, Busan 602-739, Republic of Korea

**Keywords:** stem cell-like colon cancer cells, oncolytic virus, cancer-favoring vaccinia virus, wyeth strain, resistance

## Abstract

Stem cell-like colon cancer cells (SCCs) pose a major challenge in colon cancer treatment because of their resistance to chemotherapy and radiotherapy. Oncolytic virus-based therapy has shown promising results in uncured cancer patients; however, its effects on SCCs are not well studied yet. Here, we engineered a cancer-favoring oncolytic vaccinia virus (CVV) as a potent biotherapeutic and investigated its therapeutic efficacy in terms of killing SCCs. CVV is an evolved Wyeth strain vaccinia virus (EVV) lacking the viral thymidine kinase. SCC models were established using human or mouse colon cancer spheres, which continuously expressed stemness markers. The cancer-favoring characteristics and different cytotoxic pathways for killing cancer cells successfully overrode general drug resistance, thereby killing colon cancer cells regardless of the presence of SCCs. Subcutaneously injected HT29 spheres showed lower growth in CVV-treated models than in 5-Fu-treated models. Intraperitoneally injected CT26 spheres induced tumor masses in the abdominal region. CVV-treated groups showed higher survival rates and smaller tumor mass formation, compared to 5-Fu-treated groups. Interestingly, the combined treatment of CVV with 5-Fu showed improved survival rates and complete suppression of tumor mass. The CVV developed in this study, thus, effectively suppresses SCCs, which can be synergistically enhanced by simultaneous treatment with the anticancer drug 5-Fu. Our novel CVV is highly advantageous as a next-generation therapeutic for treating colon cancer.

## INTRODUCTION

Cancer is one of the leading causes of death, with a mortality rate (deaths per 1,000 individuals per year) of approximately 14% worldwide. Among the various types of cancer, colorectal cancer (CRC) has a relatively poor prognosis and is diagnosed as the third most common cancer in men and the second in women in 2012 [1, 2]. Despite advances in anticancer drug development, most CRCs are resistant to conventional cancer therapy. This poor responsiveness to chemo- and/or radiotherapy might be attributed to stem cell-like colon cancer cells (SCCs) [3]. It is generally accepted that cancer stem cells (CSCs) are capable of self-renewal and have the exclusive ability to reproduce malignant tumors even though CSCs constitute a minor population in tumors. Drug resistance and tumor recurrence after the initial response to chemo- or radiotherapy may be due to the survival of CSCs within the original tumor. Therefore, SCCs pose a considerable challenge and are considered the next target in colon cancer treatment.

Genetic engineering of viruses provides unprecedented opportunities for various biomedical applications, including drug/gene delivery, tissue engineering, targeting cancer, and/or cancer imaging [4–11]. Oncolytic viruses (OVs) are unique biomaterials having merits over conventional anticancer reagents in terms of their tumor selectivity and ability to lyse cancer cells. Tumor selectivity is usually introduced by genetic engineering, which attenuates viral replication in normal cells. In our previous study, clinically applied JX-594 conferred tumor selectivity *via* viral thymidine kinase (vTK) inactivation because vaccinia virus has evolved to replicate in EGFR pathway-activated cells, which are usually cancer cells with high cellular TK levels [10, 12–14]. Thus, OVs can selectively infect and replicate in cancer cells. OVs are replication competent; thus, the infectious progeny generated by OV replication in tumor cells can expand to kill the tumor mass, whereas OV rarely harms normal cells. OV-based therapy in actual clinical settings began over a century ago, demonstrating the effectiveness of OVs in cancer treatment [13, 15–17]. Among them, vaccinia virus-based therapy is well tolerated and has shown relatively low side effects: minor and expected controllable toxicity and no evidence of uncontrolled or latent infection, or unexpected disease occurrence [18].

Despite the above proven efficacy of OVs in cancer cells/tissues in clinical settings, the effects of OVs on SCCs need to be investigated further. Herein, we engineered a cancer-favoring oncolytic vaccinia virus (CVV) and investigated its effects on CRC in terms of killing SCCs. We hypothesized that the cancer-favoring characteristics, cancer cell selectivity, and cancer cell infectivity mediated by vaccinia virus differ from those of conventional anti-cancer drugs; thus they may help suppress the growth of SCCs.

## RESULTS

### CVV selectively infects and kills various CRC cell lines better than VR1536

**Figure 1:**
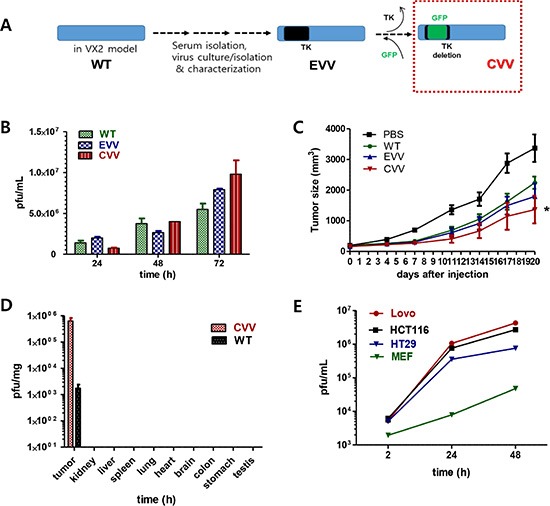
Schematic illustration of our approach to construct CVV and its higher cancer selectivity (**A**) We engineered a cancer-favoring virus (CVV) from the Wyeth strain of vaccinia virus. (**B**) Viral replication assay showing that CVV deficient of vTk replicated at lower levels at 24 h post-infection, but showed higher replication rates than EVV and WT. (**C**) CVV shows enhanced suppression of tumor size in CT26 xenograft mice compared to that observed in mice administered PBS, WT or EVV (*n* = 6, *p < 0.05 *vs.* PBS). (**D**) CVV-biodistribution results at 2 weeks post-intraperitoneal injection in HT29-bearing mice, showing that CVV selectively infected tumor mass. (**E**) CVV showed higher infectivity in colorectal cancer cell lines than in normal mouse embryonic fibroblasts.

CVV was generated by replacing the vTK gene from a naturally evolved cancer-favoring Wyeth strain vaccinia virus (EVV) strain [19] with the green fluorescence protein gene (Figure 1A). EVV was constructed from the Wyeth strain of vaccinia virus to achieve the cancer-favoring property and then isolated and characterized by repeated replication and tumor tissue lysis [19]. EVV was isolated from the blood of a vaccinia virus-injected VX2 tumor animal model when the tumor size became reduced and started to release viruses into the serum. Previously, we found that EVV had superior tumor selectivity compared with the wild type (WT) virus and other engineered vaccinia viruses [19]. CVV may work highly effectively compared to other type of virus. Replication efficacy generally reflects the antitumor activity and was examined in CT26 cells (Figure 1B). Viral replication assay results showed that CVV deficient of vTk showed lower infection at 24 h, but showed higher replication rates subsequently, compared to EVV and the WT virus. A lower initial replication of CVV likely resulted from vTk deficiency, where higher replication rates of CVV in Tk-activated host cancer cell lines are attributable to its higher tumor selectivity. Enhanced suppression of colon tumors by CVV treatment, compared to PBS, WT, or EVV administration, was confirmed in an *in vivo* CT26 xenograft model (Figure 1C). We used 10^6^ plaque-forming units (pfu) virus/mouse because CVV may have a higher replication rate than the WT virus or EVV. The infectious dose of the WT or JX594 viruses used in a previous *in vivo* study was more than 10^7^ pfu [14]. As expected, CVV infection exhibited better results than WT or EVV, even with a single injection at the low dose of 10^6^ pfu/mouse.

**Figure 2:**
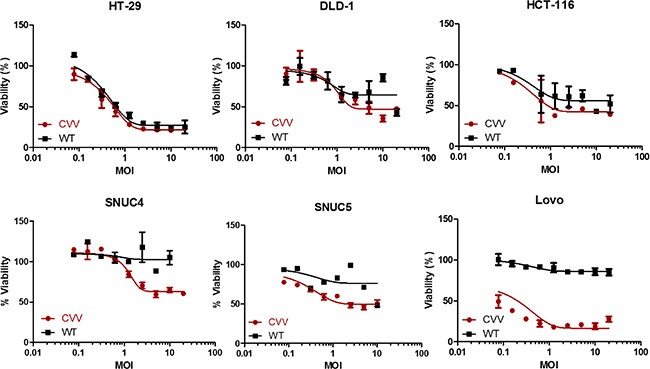
CVV selectively infected and killed various colorectal cancer cell lines more efficiently than did the WT vaccinia virus VR1536 (WT) CVV infection showed higher cytotoxicity than did the WT virus in various colorectal cancer cells (CRCs) used in the WST assay.

The higher anti-cancer efficacy of CVV in colon cancer cells is due to its greater selectivity (cancer-favoring characteristics via evolution and TK deletion). To test the tumor selectivity and safety for normal tissues, tumor tissues and normal tissues (kidney, liver, spleen, lung, heart, brain, colon, stomach, and testis) were harvested at 2 weeks after intraperitoneal injection of HT29 cells into nude mice, and virus titers in each tissue (pfu/mg) confirmed that selective infection of tumor tissues occurred (Figure 1D). We found marginal viral replication (∼5 pfu/mg) in normal kidney and liver tissues, and no viral replication in normal spleen, lung, and colon tissues in the biodistribution data, which was is marked contrast with viral titers found in tumor tissues (∼10^6^ pfu/mg). The higher toxicity of CVV in cancer cells than in mouse embryonic fibroblasts (MEFs), used as normal cells might be ascribed to its higher infectivity in CRC cell lines (Figure 1E), and the fact that the CVV infectivity was much lower than that in cancer cell lines. The reason why we found viral replication in MEF cell lines, but not in normal tissues, in the biodistribution data may be because selectivity can be generally measured only when a virus has the option to infect either normal or tumor tissues. Within MEF cells, viral replication should occur in normal cells (given no other choice) by utilizing the replication properties of the cell lines. Therefore, higher infectivity in cancer cell lines than that in normal cells can explain why CVV selectively distributed to tumor tissues. To demonstrate the cancer-favoring and oncolytic potency of CVV in colon cancer, a panel of CRC cell lines (HT-29, DLD-1, HCT-116, SNU4, SNU5, and/or Lovo) was tested. Following infection, CVV showed higher toxicity than the WT virus in the various CRC cell lines (Figure 2).

### CVV might overcome resistance observed in stem cell-like cancer cell populations

**Figure 3:**
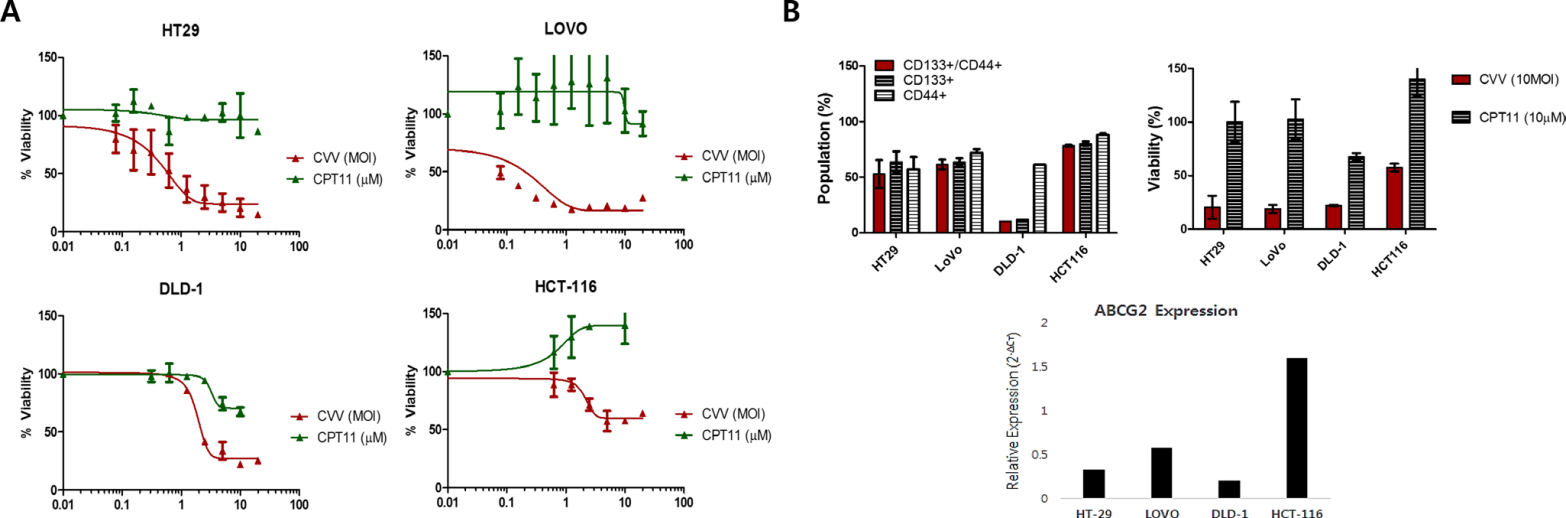
Cytotoxicity of CVV towards CPT11-resistant cells may overcome the stem cell-like cancer cell population (**A**) The percent viability of 4 different colorectal cancer cells (CRCs) shows their resistance to CPT11 exposure, but not to CVV infection. (**B**) The percentage of the CD133^+^/CD44^+^ double-positive cell population, which represents SCCs, with CD133^+^ and CD44^+^ cells (left panel) and the percent viability of each CRC following CVV infection (MOI = 10) and CPT11 treatment (10 μM) (right panel). The relative expression of ABCG2 (bottom panel) shows its relationship to drug resistance and the SCC population.

CVV infection showed greater toxicity than that of the anti-colon cancer drug CPT11 in CRCs (HT29, LOVO, DLD-1, and HCT-116), as shown in Figure 3A. The units used for CVV and CPT11 treatment are the multiplicity of infection (MOI) and the micromolar concentration, respectively. Although a direct comparison of the viral MOI and CPT11 concentration is impossible, it is safe to assume that the virus concentration used was much lower than CPT11 concentration used when considering their approximate molar concentrations. In addition, all of the cell lines tested showed resistance to CPT11, even up to doses of ∼10 μM (Figure 3A). We then analyzed the percentage of the CD133^+^/CD44^+^ double-positive cell population, which represents SCCs, and its relation to drug resistance. The population of SCCs is shown in Figure 3B (left panel). The percent viability of each CRC after CVV infection (MOI = 10) or CPT treatment (10 μM) is shown in the right panel of Figure 3B. Interestingly, the percentage of the CD133^+^/CD44^+^ population appeared to be related with CPT11 resistance, whereby the CD133^+^ population seemed to majorly confer the percentage of CD133^+^/CD44^+^ SCCs and, thus, drug resistance. In terms of the percent cell viability of CPT11-treated HCT-116 cells, we observed fluctuations in the WST-1 results, which exceeded 100% compared to the viability of untreated cells (Figure 3A). The observation of elevated cell viability of HCT-116 cells (over 100%) after the 72 h-treatment may have been because WST-1 measures dehydrogenase activity, which is generally elevated in cancer stem cells. We expect that higher SCC populations remaining after CPT11 treatment would give an enhanced signal, resulting in measured cell viabilities of over 100%. The cytotoxicity of CVV towards CPT11-resistant cells may overcome the stem cell-like cancer cell population. The relative expression of ABCG2 also demonstrated the relationship between drug resistance and the SCC population (Figure 3B bottom). Thus, CPT11 resistance might be related to the SCC population and ABCG2 expression, whereas CVV showed dose-dependent responses regardless of the SCC population, suggesting that our CVV might overcome drug resistance originating from the stem cell-like colon cancer cell population.

### Stem cell-like colon cancer cells have sphere-forming ability and highly tumorigenic, but are more susceptible to CVV than to CPT11

**Figure 4:**
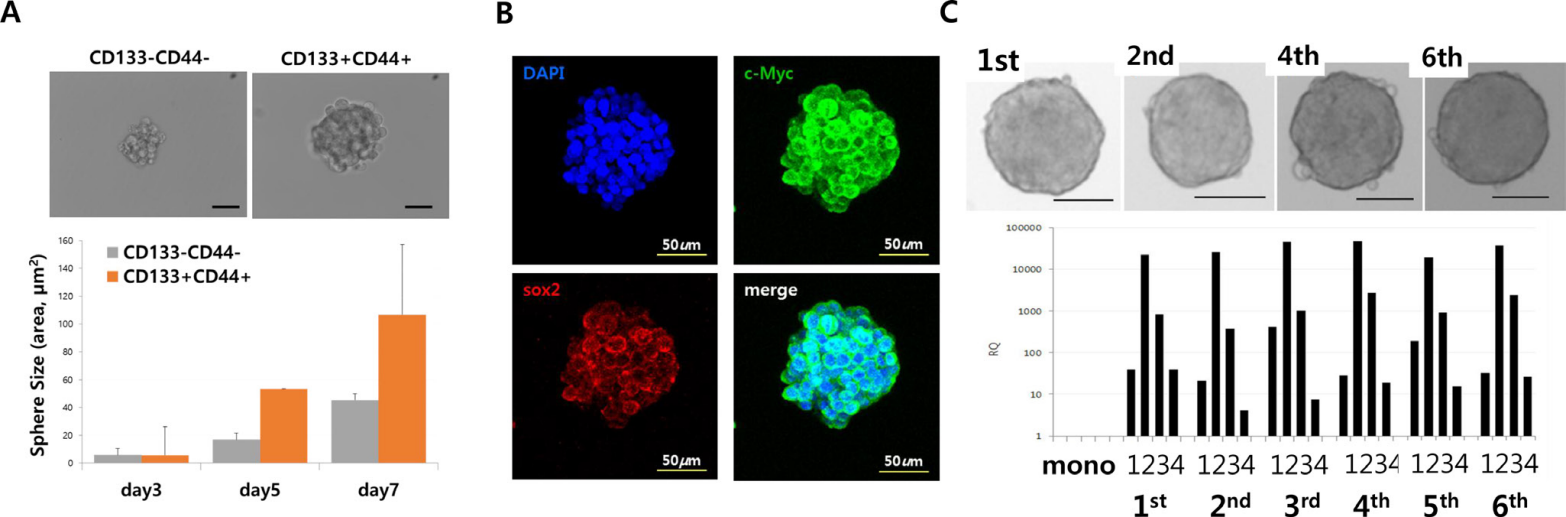
Stem cell-like cancer cells have sphere-forming ability (**A**) Sphere-forming ability of CD133/CD44^−^ and CD133^+^/CD44^+^ cells (upper panels). Sphere size of CD133−/CD44^−^ and CD133^+^/CD44^+^ cells (bottom panels). (**B**) Immunostaining of stemness markers c-Myc and Sox2 in HT29 sphere cells. (**C**) Sequential sphere-forming ability and continuous stemness maker expression (Scale bar = 100 μm; upper panels). Relative expression of the stemness markers Nanog (1), Sox2 (2), Oct4 (3), and c-Myc (4) in CD133^−^/CD44^−^ and CD133^+^/CD44^+^ cells (bottom panel).

Three-dimensional sphere cultures of tumor cells lead to the enrichment of CSC populations [20]. To confirm the relationship between sphere-forming ability and cell stemness, CD133^+^/CD44^+^ and CD133^−^/CD44^−^ HT29 cells were analyzed after fluorescence-activated cell sorting (FACS) and compared with unsorted HT29 cells. As expected, double-positive (CD133^+^/CD44^+^) HT29 cells showed better sphere-forming ability than did CD133^−^/CD44^−^ cells (Figure 4A). The sphere size of CD133^+^/CD44^+^ cells increased compared to CD133^−^/CD44^−^ over sequential days in culture. Immunostaining of HT29 spheres confirmed that they expressed stemness marker proteins (Figure 4B). When 5–7-day-old cultured spheres were dissociated and re-cultured in stem cell media, HT29 spheres could be formed repeatedly up to 6 or more times in sequential sphere cultures. In addition, repeated sphere formation/dissociation did not impair the sphere-forming ability or stemness gene expression for up to 6 days (Figure 4C), demonstrating that sphere formation contributes to the characteristics of stemness of cancer cells. Different numbers of monolayer-cultured and sphere-cultured HT29 cells (5 × 10^3^, 1 × 10^4^, and 5 × 10^4^) were injected subcutaneously into Balb/c nude mice, after which their tumorigenicity (Figure S1A) and tumor volumes (Figure S1B) were examined As expected, HT29 spheres showed characteristics of SCCs in a mouse *in vivo* model, as earlier tumor formation and larger tumor volumes were observed with the group injected sphere-cultured cell than the group administered monolayer-cultured cell.

### Enhanced suppression of stem cell-like colon cancer cells by CVV may overcome anticancer drug-resistance in SCCs

**Figure 5:**
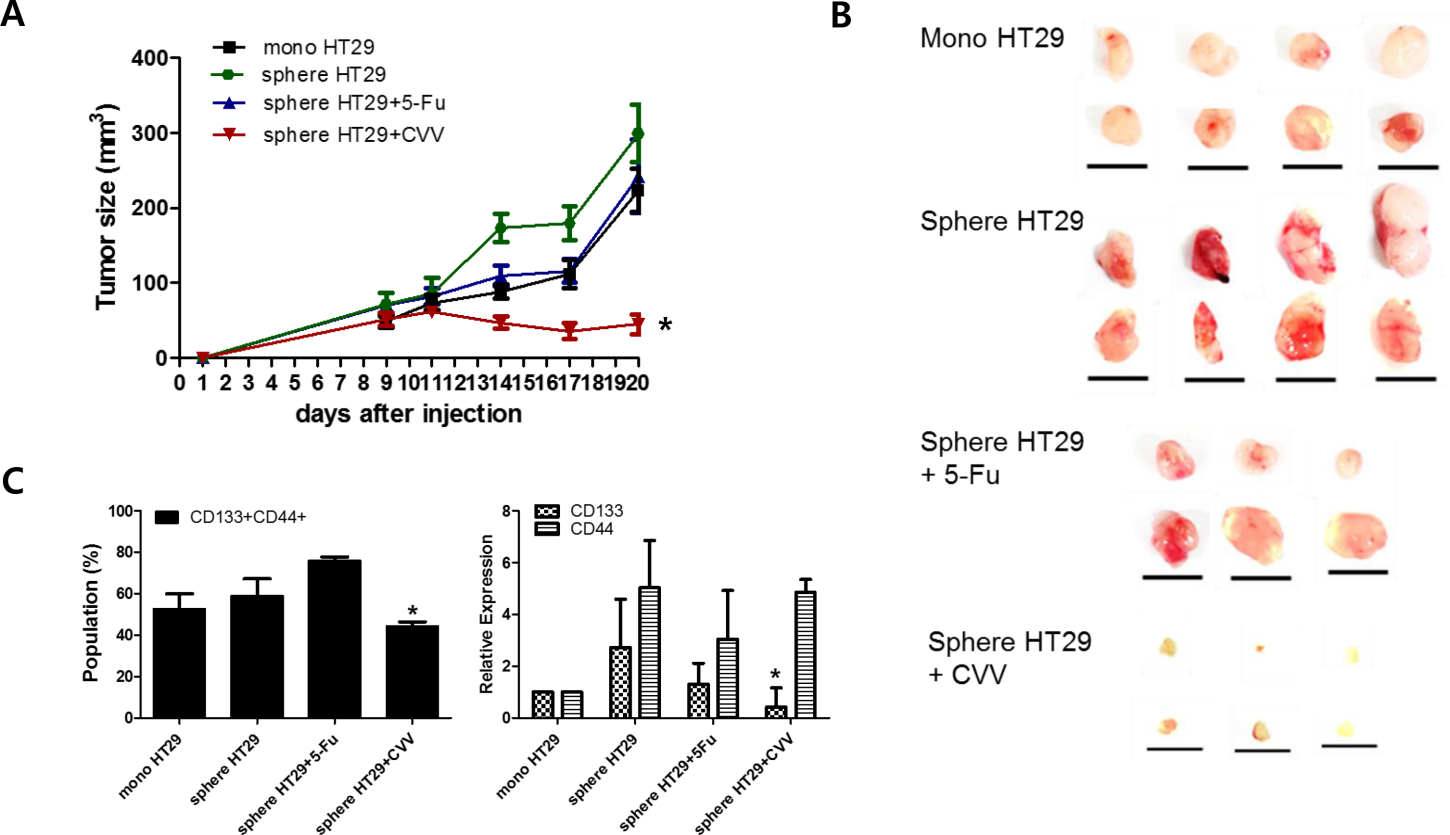
Enhanced suppression of stem cell-like HT29 human colon cancer cells by CVV (**A, B**) Tumor sizes measured in xenograft models of 10^6^ live monolayer-cultured HT29 cells (mono HT29), sphere-cultured HT29 cells (sphere HT29), sphere HT29 cells treated with 5-Fu, and HT29 cells treated with CVV showed that CVV can attenuate tumor growth and successfully kill stem cell-like cells (*n* = 6–8; *p < 0.05 *vs.* mono HT29, *t* test; scale bar = 1 cm). (**C**) The CD133^+^/CD44^+^ double-positive SCCs population (left panel) and real-time PCR results (right panel) show that CVV can highly suppress stem cell-like HT29 cells compared to 5-Fu treatment (*n* = 3, *p < 0.05).

We then cultured HT29 spheres for 5 days, treated them with 0.1 μM Fluorouracil (5-Fu) or CVV (MOI = 0.1), and harvested them after an additional 3 days in culture. Monolayer-cultured HT29 cells (mono HT29) and sphere-cultured HT29 cells (sphere HT29) were also harvested. Live cells (1 × 10^6^), including mono HT29s, sphere HT29s, sphere HT29s treated with 5-Fu (sphere HT29s+5-Fu), and HT29s infected with CVV (sphere HT29s+CVV), were injected subcutaneously into Balb/c nude mice, and tumor growth from the injected surviving cells was examined after treatment (Figure 5). As expected, animals injected with HT29 spheres having the characteristics of SCCs showed earlier and larger tumor growth *versus* animals injected with monolayer-cultured cells. Animals with surviving HT29 sphere cells after CVV treatment showed attenuated tumor growth resulting from successful killing of stem cell-like cells by CVV, whereas surviving HT29 sphere cells following 5-Fu treatment did not (Figure 5A & 5B). FACS analysis (Figure 5C, left panel) and real-time PCR results (Figure 5C, right panel) showed that the CD133^+^/CD44^+^ double-positive cell population increased or decreased after 5-Fu or CVV treatment, respectively, suggesting that CVV infection could kill stem cell-like HT29 cells more so that 5-Fu treatment. Thus, surviving cells after 5-Fu treatment showed much higher levels of SCC marker expression. Therefore, cancer-favoring natural and engineered vaccinia viruses have more powerful cancer selectivity and higher cytotoxicity. The underlying mechanism, however, differs from that of anticancer drugs, thereby obviating the problem of resistance.

### Enhanced suppression of stem cell-like CT26 mouse colon cancer cells by CVV in a syngeneic model of CRC peritoneal carcinomatosis

**Figure 6:**
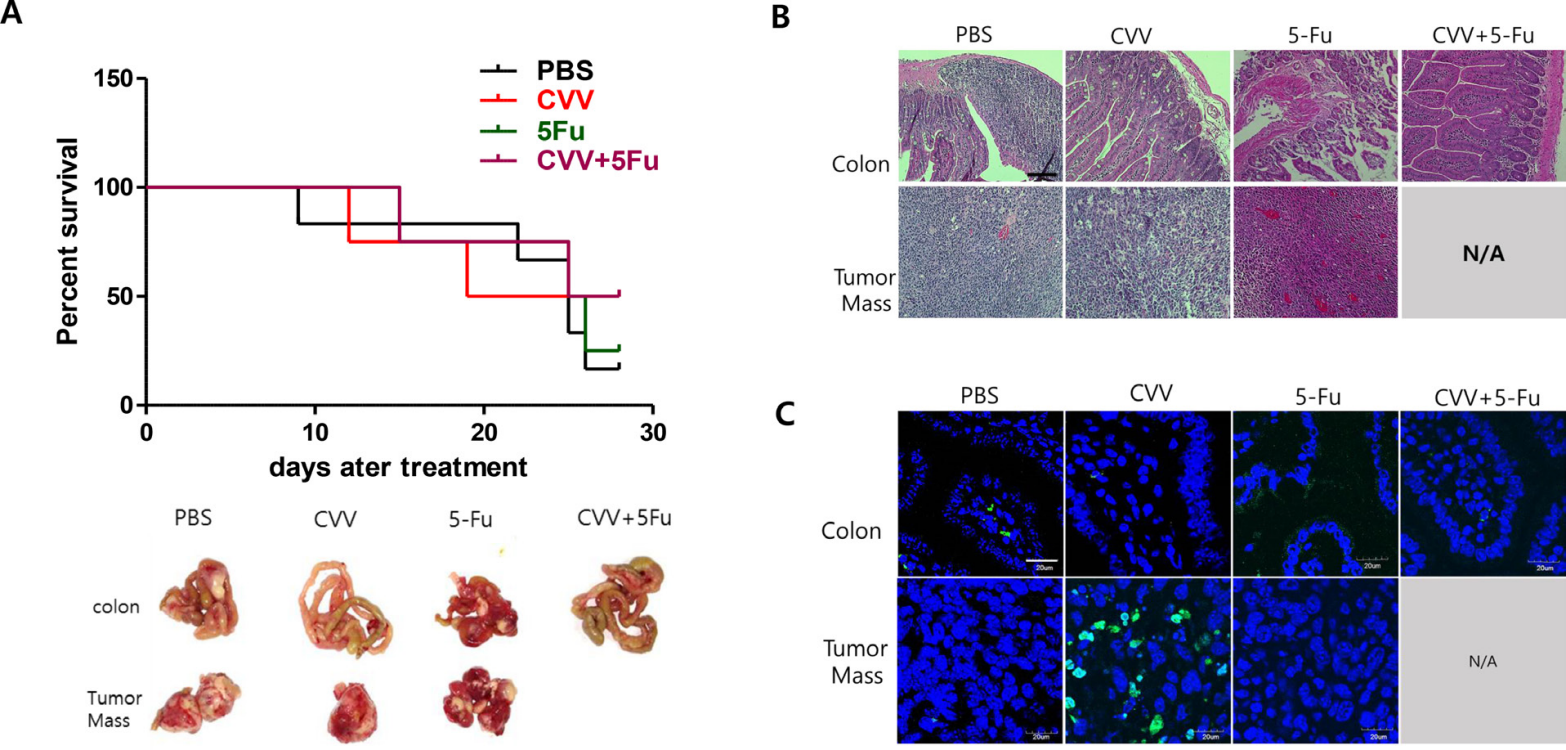
Enhanced suppression of stem cell-like CT26 mouse colon cancer cells by CVV (**A**) Percent survival rates of mice intraperitoneally injected with CT26 spheres and treated with PBS, CVV, 5-Fu, or CVV+5-Fu (*n* = 4–6). (**B**) H & E staining results of colon tissues and tumor masses of mice intraperitoneally injected with CT26 spheres and treated with PBS, CVV, 5-Fu, or CVV+5-Fu. (**C**) TUNEL staining results of colon tissues and tumor masses of mice intraperitoneally injected with CT26 spheres and treated with PBS, CVV, 5-Fu, or CVV+5-Fu (Scale bar = 20 μm).

**Figure 7:**
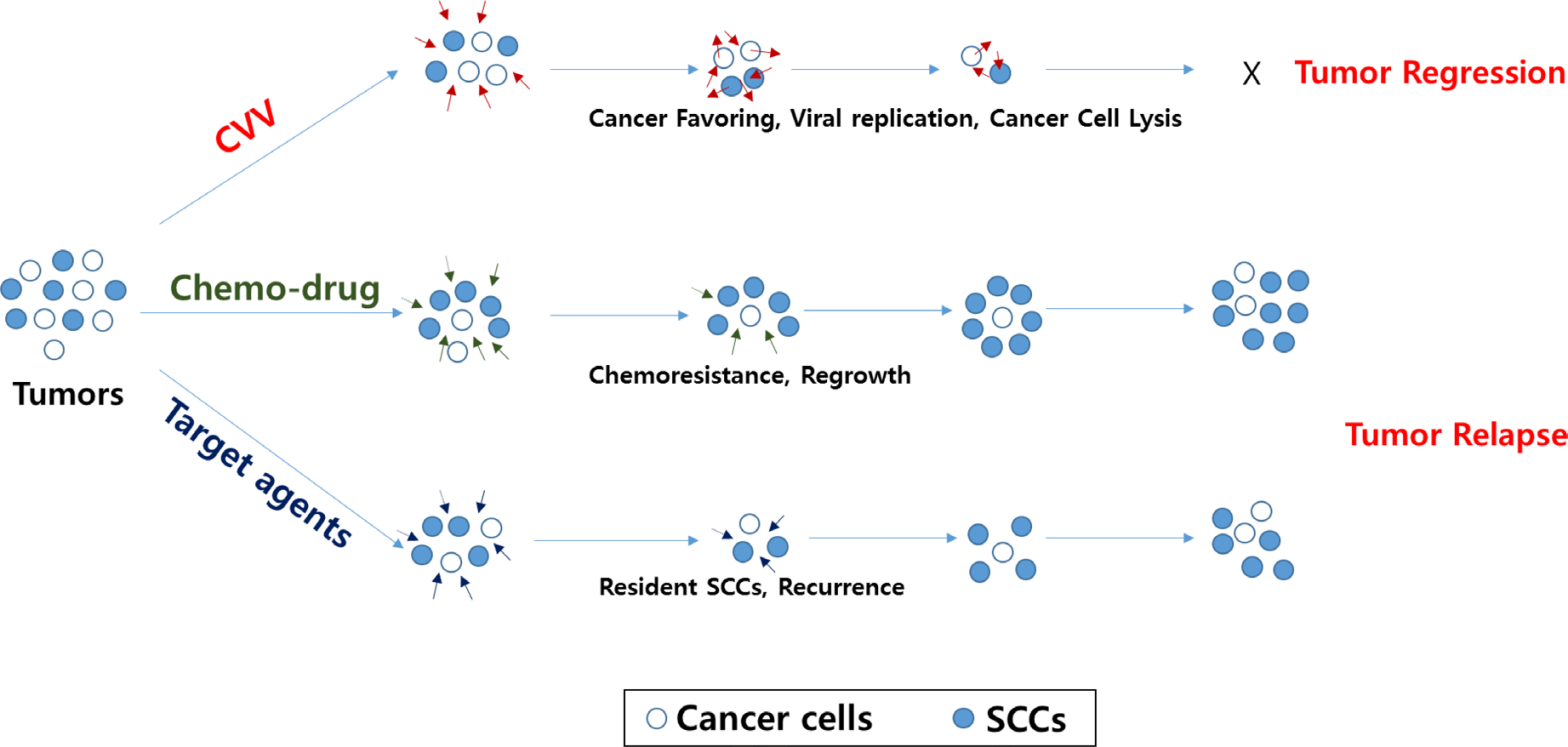
Schematic illustration of how CVV can kill colon cancer cells by overcoming SCC issues The CVV has enhanced cancer-favoring selectivity and cytotoxicity, thereby enabling it to successfully override drug resistance and/or other limitations of currently developed targeted therapies in terms of killing colon cancer cells, regardless of the presence of SCCs, because of its cancer-favoring characteristics and the different cytotoxic pathway used to kill cancer cells.

The CT26 mouse colon cancer cell line was chosen for an *in vivo* syngeneic carcinomatosis mouse model with SCCs. Percent survival rates of the models intraperitoneally injected with CT26 spheres and treated with PBS, CVV, 5-Fu, or CVV+5-Fu were in line with our expectations (Figure 6A). Comparison of the efficacy of 5-Fu and CVV treatment on the survival showed that the 5-Fu group had good survival until 24 days post-treatment; however, CVV-treated mice showed a longer survival period. Interestingly, combined treatment with CVV and 5-Fu improved the survival rate compared to the 3 other groups. At 30 days post-treatment, the surviving mice were sacrificed, and the internal peritoneal regions were examined. Intraperitoneally injected sphere-cultured CT26 cells spread into the liver and also connected the tumor mass with colon tissues in the abdomen (in the PBS model). Interestingly, no tumor masses were found in the abdomens of the co-treatment group (CVV+5-Fu) (Figure 6A, bottom). Hematoxylin and eosin (H & E) staining results of colon tissues and tumor masses of CT26 sphere-injected models treated with PBS, CVV, 5-Fu, or CVV+5-Fu revealed that fewer inflammatory and tumor regions were found in the CVV-treatment and co-treatment groups than in the PBS- and 5-Fu-treatment groups (Figure 6B). Importantly, terminal deoxynucleotidyl transferase dUTP nick end labeling (TUNEL) staining results of colon tissues and tumor masses of CT26 sphere-injected models treated with PBS, CVV, 5-Fu, or CVV+5-Fu indicated that CVV effectively induced apoptosis only in the tumor mass (Figure 6C). The results showed that SCCs can respond better to CVV than the anticancer drugs CPT11 or 5-Fu, particularly for longer periods. We propose that this might be because anticancer drugs are sufficiently cytotoxic to kill general cancer cells, and thus, better results in the short-term are exhibited. However, resistant SCCs can eventually emerge, although the effect of CVV infection can override the effect of resistance to anticancer drugs (Figure 7).

## DISCUSSION

SCCs are tumorigenic and are responsible for the malignancy and drug resistance of colon cancer cells even though SCC populations constitute only 0.1–10% of all tumor cells [21]. The rare subset of cancer cells having “stem-like” features, namely, CSCs, possess natural quiescence and a high level of drug efflux transporters, leading to their low sensitivity to chemotherapy and radiotherapy, compared with normal tumor cells [21–24]. Therefore, the concept has emerged that CSCs represent an important challenge in the understanding of anticancer drug resistance, cancer recurrence, and metastasis [25], as well as CRC treatment. The available conventional therapeutic agents primarily eliminate the bulk of tumors, but do not affect CSCs [26, 27]. Therefore, the ability to target and eradicate CSCs is expected to markedly improve clinical outcomes. The most common way of identifying and isolating CSCs is based on cell surface marker expression using cell-separation methods, such as flow cytometry, magnetic sorting, or FACS. Surface markers for different types of CSCs in brain [28], breast [29], ovarian [30], colon [20, 31], lung [32], head and neck [33], pancreas [34], and liver cancers [35] have been identified. Other ways to identify CSCs include detecting the expression of transcription factors and various cytoplasmic and nuclear proteins, and/or determining functional properties such as DNA-label retention, dye-efflux properties, or enzymatic metabolism. However, no single marker can define CSCs, but the combined use of multiple markers now offers the potential to identify and define CSCs. Indeed, CSCs share many characteristics with normal stem cells [36]; therefore, developing specific agents for targeting and killing CSCs warrants further research.

Recently, OV-based therapy has attracted attention because it shows promising results in terms of cancer selectivity and safety in clinical trials [37–40]. OVs possess cancer selectivity and efficiently kill cancers because (i) OVs utilize the EGFR pathway, which is activated in cancerous cells; (ii) elevated TK in cancer cells promotes enhanced replication of vTk-deleted OVs; (iii) OV kills cancer by replicating within the cancer, so that it can be effectively used at lower concentrations to stop replication when all the host cancer cells are lysed; and (iv) cancer cells lysed by an OV can release debris containing tumor antigens, which triggers the immune system and leads to cytotoxic T-cell activation and cell-mediated immunity [41]. Although encouraging results from clinical trials are beginning to accumulate, the efficacy of using OVs for SCCs has not been well understood thus far. OVs can be ideal candidates, as opposed to directly targeting agents for CSCs, because they are cytotoxic and are not subject to the typical mechanisms of drug resistance, such as drug-efflux pumps and defective apoptotic signaling, which suggests that OVs might be effective against CSCs, including SCCs [42, 43].

In this study, efforts were made to develop CVV, a cancer-favoring oncolytic vaccinia virus, by first evolving vaccinia virus from the tumor mass and then inactivating the vTk gene. We next investigated its therapeutic efficacy in terms of killing SCCs. When applied to various CRC cell lines, CVV could selectively infect and kill host cells more efficiently than the wild-type vaccinia virus VR1536. However, most cells showed anticancer drug resistance, which is associated with SCC populations and are related with ABCG2 expression. SCC models were established by constructing colon cancer sphere cell lines, which have higher tumorigenicity and can continuously express stemness markers. As expected, sphere cells remained susceptible to CVV, but not to chemodrugs. Our CVV also selectively killed tumor cells in an *in vivo* mouse model. Tumor xenografts induced by subcutaneous injection with HT26 spheres that survived after pretreatment with PBS, CVV, or 5-Fu showed significantly suppressed tumor growth, only with CVV treatment. Interestingly, 5-Fu showed an early response in intraperitoneally injected CT26 mice in terms of suppressing tumor masses and increasing the survival rates of treated mice, but it was surpassed by CVV 24 at days post-treatment. Our data also showed that CVV eventually exhibited greater anti-tumor efficacy, even after a single, low-dose injection (10^6^ pfu/mouse). Combined treatment with CVV and 5-Fu further improved survival rates and led to complete suppression of tumor mass production in the abdomen. We ascribe this finding to the early effect of 5-Fu, followed by the late effect of CVV owing to its replication and mild effect in cancerous tissue. The early effect of 5-Fu can kill bulk cancer cells, but SCCs can retain resistance. This issue, however, was counteracted by CVV. Furthermore, the immune reaction conferred by cancer cells lysed by CVV may also contribute to the relatively long-lasting effect of suppressing CSCs.

## CONCLUSIONS

The CVV developed in this study can effectively and selectively infect and replicate in SCCs because it favors cancer cells and is not affected by drug-resistance pathways. We conclude that this CVV effectively suppressed SCCs, which can be synergistically enhanced by co-treatment with 5-Fu.

## MATERIALS AND METHODS

### Cell culture and reagents

The human colon cancer cells lines HT29, Lovo, DLD-1, and HCT116, and the mouse colon cancer cell line CT26 were obtained from the Korean Cell Line Bank (Seoul, Korea) and the ATCC (Manassas, VA). Cells were cultured in RPMI-1640 supplemented with 10% FBS and 100 U/mL penicillin and streptomycin under standard conditions of 37°C, 5% CO_2_, and a humidified atmosphere. All culture media and supplements were from Welgene (Daegu, Korea). The anticancer drugs CPT11 and 5-Fu were purchased from Sigma Korea (Yongin, Korea).

### Sphere formation

Single cells were resuspended in serum-free DMEM/F12 (Welgene) containing 1 × B27 supplement (Invitrogen, Waltham, MA), 20 ng/mL epidermal growth factor, and 10 ng/mL basic fibroblast growth factor (PeproTech, Rocky Hill, NJ). Primary spheres were derived by plating 50,000 to 100,000 single cells per well into 6-well ultra-low attachment dishes (Corning, Corning, NY). Dishes were cultivated for 7 days prior to *in vivo* cell injection.

### Cell proliferation (cytotoxicity) assay

Cells were seeded at 10,000 cells per well in 96-well plates. After 1 day, cells were treated with an anticancer drug or OV at the desired concentration in serum-free media for 2 h. Then, the media were replaced with normal culture media. After a 72-h treatment, cell viability was assessed using the WST Cell Proliferation Assay (EzCytotox, ITSBIO, Seoul, Korea) according to the manufacturer's instructions. The absorbance of each sample at 450 nm was measured using a microplate reader. The reference wavelength used was 680 nm.

### Replication assay

Viral replication was quantified using a standard plaque assay. Cells were plated at 40,000 cells in 24-well flat-bottom plates in 1 ml of media and incubated at 37°C. Cells were infected with the virus of interest (WT, EVV, or CVV) at an MOI of 0.1. Cell lysates were harvested daily for 3 days and viruses were released by 3 cycles of freezing and thawing of the cell lysates. Serial dilutions of each sample were used to infect U2OS cells at 80% confluency in 6-well plates, and viral titers were determined by counting viral plaques.

### Real-time PCR

Total RNA was extracted using the TRIzol reagent (Invitrogen). RNA purity was verified by measuring the ratio of absorbance at 260 and 280 nm (A260/280). The first strand of cDNA was synthesized with 2 μg of total RNA using the PrimeScript 1st Strand cDNA Synthesis Kit (Takara Korea, Seoul, Korea), and 1 μL of the cDNA was used for each PCR mixture containing SYBR-Green qPCR mix (Roche, BASEL, Switzerland). Real-time PCR was performed using a LightCycler 96 Real-Time PCR System (Roche). The reaction mixtures were subjected to a 40-cycle amplification at 95°C for 20 s, 60°C for 20 s, and 72°C for 25 s. Relative mRNA expression levels of the selected genes were normalized to beta-actin mRNA expression and quantified using the 2^−ΔΔCt^ method. The sequences of the primers used are shown in Table 1.

**Table 1:** Sequences of primers used in this study

Name	Sequences (5′→3′)	Product size (bp)
Nanog Forward	CCT GAT TCT TCT ACC AGT CCC A	123
Nanog Reverse	GGC CTG AGA GAA CAC AGT CC	
Sox2 Forward	GCA CAT GAA CGG CTG GAG CAA CG	207
Sox2 Reverse	TGC TGC GAG TAG GAC ATG CTG TAG G	
Oct4 Forward	ATG TTT CTG AAG TGC CCG AA	85
Oct4 Reverse	AGA GAA GGA TGT GGT TCG AG	
cMyc Forward	TGA CCT AAC TCG AGG AGG AGC TGG AAT C	170
cMyc Reverse	AAG TTT GAG GCA GTT AAA ATT ATG GCT GAA GC	
β-Actin 184 Forward	AGA GCT ACG AGC TGC CTG AC	184
β-Actin 184 Reverse	AGC ACT GTG TTG GCG TAC AG	

### Flow cytometry

FACS analysis and cell sorting were performed using a FACSCanto II and FACSAria II (Becton Dickinson, Franklin Lakes, NJ), respectively. FACS data were analyzed using FACS Diva software (BD). Antibodies to the following proteins were used: FITC-conjugated CD44 (MACS, Miltenyi Biotech Korea, Seoul, Korea) and PE-conjugated CD133 (BD). FACS gates were established by staining with isotype-matched PE or FITC conjugated antibodies (BD).

### Immunofluorescence staining

Samples were fixed with 4% paraformaldehyde for fluorescence staining. Samples were permeabilized with 0.1% Triton X-100, and nonspecific binding was blocked with 5% normal goat serum (Invitrogen). Staining was performed using unlabeled primary antibodies and fluorophore-conjugated secondary antibodies. Stained samples were examined by fluorescence confocal microscopy (Olympus IX7; Hicksville, NY).

### Animal study

All mice were maintained according to protocols approved by the Institutional Animal Care and Use Committee of the Pusan National University (PNU-2014-06850). Nude mice and BALB/c mice were purchased from Orient (Gapyeong, Korea) and HANA (Pusan, Korea). Colon cancer cell lines (10^5^−10^6^ cells) were injected into 6-week-old male BALB/c mice via subcutaneous or intraperitoneal injection. Tumor sizes were measured twice per week in the subcutaneous injection model. The tumor volume was calculated according to the following formula: tumor volume (mm^3^) = L × W^2^/2, where L is the tumor length and W is the tumor width. The animals in the intraperitoneal injection model were divided into 4 groups, which received intraperitoneal injection of PBS, 5-Fu (25 mg/kg, 3 times a week), CVV (1 × 10^6^ pfu/mouse, once a week), and 5-Fu (25 mg/kg, 3 times a week) plus CVV (10^6^ pfu/mouse, once a week). Mouse survival was monitored daily during the experimental period. At 4 weeks post-injection, the mice were sacrificed and tissues were immediately fixed in 4% paraformaldehyde.

### H & E staining and TUNEL assay

To assay tumor generation and morphological characteristics, frozen tumor sections were used for H&E staining. Images were acquired using an Olympus confocal microscope. The TUNEL assay was performed on paraffin-embedded sections. Commercially available reagents (DeadEnd Fluorometric TUNEL System; Promega, Madison, WI) were used to perform the TUNEL analysis.

## SUPPLEMENTARY MATERIALS FIGURE


