# Supplementary material for: A cancer-favoring oncolytic vaccinia virus shows enhanced suppression of stem-cell like colon cancer
Source: Oncotarget. 2016 Feb 24;7(13):16479–89. doi: 10.18632/oncotarget.7660 (PMC4941329; doi:10.18632/oncotarget.7660)
Supplement: Supplementary file 1 [file oncotarget-07-16479-s001.pdf]

A cancer-favoring oncolytic vaccinia virus shows enhanced suppression of stem-cell like colon cancer

Supplementary Materials

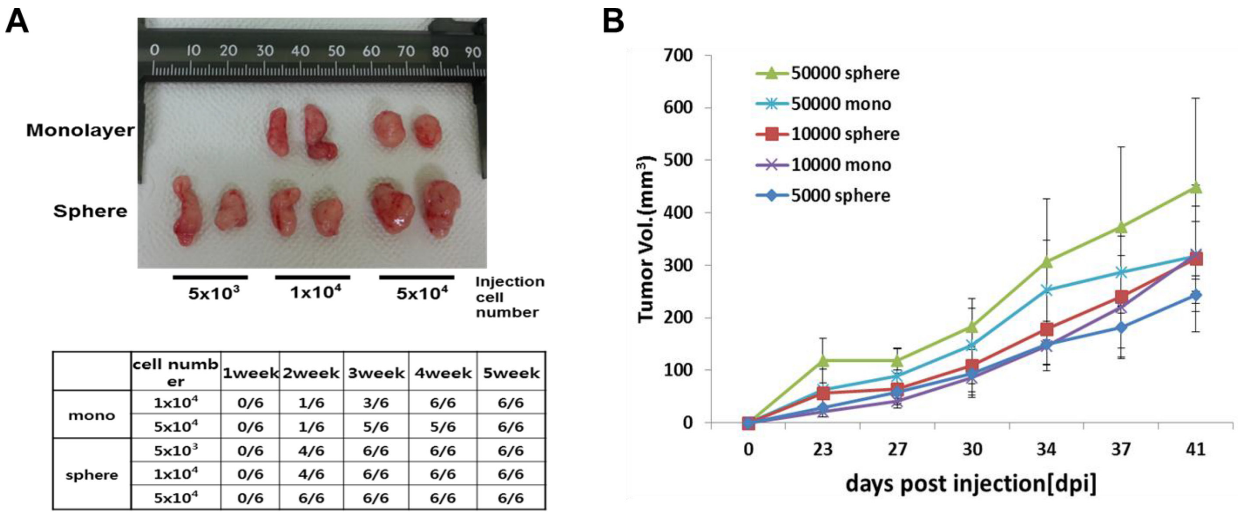

	cell number	1week	2week	3week	4week	5week
mono	$1x10^4$	0/6	1/6	3/6	6/6	6/6
	$5x10^4$	0/6	1/6	5/6	5/6	6/6
sphere	$5x10^3$	0/6	4/6	6/6	6/6	6/6
	$1x10^4$	0/6	4/6	6/6	6/6	6/6
	$5x10^4$	0/6	6/6	6/6	6/6	6/6

Supplementary Figure S1: The sphere-forming ability relates to cancer stemness and tumorigenicity. (**A**) Tumorigenicity of different numbers of monolayer or sphere HT29 cells in the xenograft at 5 weeks after injection. (**B**) Tumor volumes formed by injection with different numbers of monolayer or sphere HT29 cells in xenografts.
